# Targeting of Periprosthetic Muscles for the Ultrasonographic Screening of Hip Abnormalities in Hip Resurfacing Arthroplasty Patients

**DOI:** 10.3390/jcm12082871

**Published:** 2023-04-14

**Authors:** Hyonmin Choe, Naomi Kobayashi, Koki Abe, Yuta Hieda, Hiroyuki Ike, Ken Kumagai, Kazuma Miyatake, Takahiro Fujisawa, Yutaka Inaba

**Affiliations:** 1 Department of Orthopaedic Surgery, Yokohama City University, Yokohama 236-0004, Japankumagai@yokohama-cu.ac.jp (K.K.);; 2 Department of Orthopaedic Surgery, Yokohama City University Medical Center, Yokohama 236-0027, Japan

**Keywords:** ultrasonography, resurfacing, arthroplasty, pseudotumor, hip pain

## Abstract

Background: Hip resurfacing arthroplasty (HRA) patients require subsequent annual screening for postoperative complications. Ultrasonography may be useful for this purpose but lacks a screening protocol for hips. The purpose of this study was to evaluate the accuracy of ultrasonography for detecting postoperative complications in HRA patients using a screening protocol that specifically targets periprosthetic muscles. Methods: We enrolled 45 hips from 40 HRA patients with a mean follow-up period of 8.2 years. MRI and ultrasonography scans were simultaneously conducted at follow-up. The ultrasonography assessments were conducted on the anterior part of the hip that targets iliopsoas, sartorius, rectus femoris, lateral with anterior superior and inferior iliac spine (ASIS and AIIS) as bony landmarks, and the lateral and posterior parts that target fascia tensor, short rotators, and gluteus minimus, medius, and maximus with greater trochanter and ischial tuberosity as bony landmarks. The accuracy of diagnosing postoperative abnormalities and the visibility of periprosthetic muscles were compared between these two modalities. Results: Both MRI and ultrasonography detected an abnormal region in eight cases comprising two infections, two pseudotumors, and four patients with greater trochanteric bursitis. Among these cases, four hips required implant removal. The increase in anterior space, measured as the distance between the iliopsoas and resurfacing head, was a good indicator for the abnormal mass in these four HRA cases. In the assessment of periprosthetic muscles, MRI showed a much lower visibility than ultrasonography in the iliopsoas (6.7% vs. 100%), gluteus minimus (6.7% vs. 88.9%), and short rotators (8.8% vs. 71.4%) due to implant halation. Conclusions: By targeting periprosthetic muscles, ultrasonography can detect postoperative complications as effectively as MRI assessments in HRA patients. Ultrasonography has superior visibility in the periprosthetic muscles of HRA patients, indicating its utility for the screening of small legions in these cases which may not be visible by MRI.

## 1. Introduction

Total hip arthroplasty (THA) is an effective surgical treatment for painful hip disorders with good clinical short- and long-term outcomes. However, there is always a risk of several postoperative complications, for instance, infections, dislocations, and adverse reaction metal debris (ARMD) [1]. Metal-on-metal (MOM) THA methods, including hip resurfacing arthroplasty (HRA), have been favorable surgical options in the past but have had a high complication rate due to ARMD, which can have devastating effects [2,3,4,5,6]. Although incidence reports with HRA are much lower than MOMTHA, annual examinations and screening for asymptomatic pseudotumor and periprosthetic joint infections (PJI) are required in all HRA patients in terms of health and economic viewpoints [7,8,9,10,11].

Measurements of metal ions are a recommended screening method for possible ARMD but can be prohibitively expensive if not covered by national health insurance [12]. MRI or CT scans are universal imaging modalities for the screening of pseudotumors [7,8,9,13,14,15,16]. However, implant halation prevents the careful assessment of periprosthetic soft tissues with these methods, which is problematic in the case of implant-related complications that mainly originate in these tissues. Recent improvements to the resolution in ultrasonography have enabled a more precise evaluation of various parts of the body and the utility of this approach has been demonstrated for the screening of ARMD in MOMTHA patients [7,8,9,16]. The advantages of ultrasonography in these instances include its cost effectiveness, availability, rapidity, non-use of radiology, and minimal effects on metal implants. On the other hand, a major drawback of ultrasonography for the screening of ARMD is the lack of an established protocol in hip joints.

HRA-related complications originate in periprosthetic soft tissues. Therefore, we utilized a standardized ultrasonography protocol for the assessment of periprosthetic muscles at the annual follow-up screen in HRA patients, and compared the findings with those obtained by MRI in our current study. The purpose of our investigation was to evaluate the accuracy of ultrasonography on the targeting of periprosthetic muscles for the detection of such abnormalities, including ARMD in HRA patients.

## 2. Materials and Methods

### 2.1. Patients

This retrospective study was approved by our institutional ethics review board (2019-322). Patients who did not wish to participate or were unable to undergo either MRI or ultrasound were excluded from this study. Among the 76 hips in 64 patients who underwent HRA between 2009 and 2015 at our hospital, 45 hips in 40 patients attended regular follow-ups for more than 3 years and were thus eligible for a comparison of imaging findings between MRI and ultrasonography for possible prosthetic abnormalities. All 40 patients underwent an MRI assessment every 3–5 years and an annual ultrasound. An ultrasound was conducted within 3 months of an MRI assessment in each subject. Among the 40 patients (45 hips) that were analyzed, 4 patients (4 hips) required revision surgery due to ARMD or periprosthetic joint infection (PJI).

### 2.2. Ultrasonography

**Figure 1:**
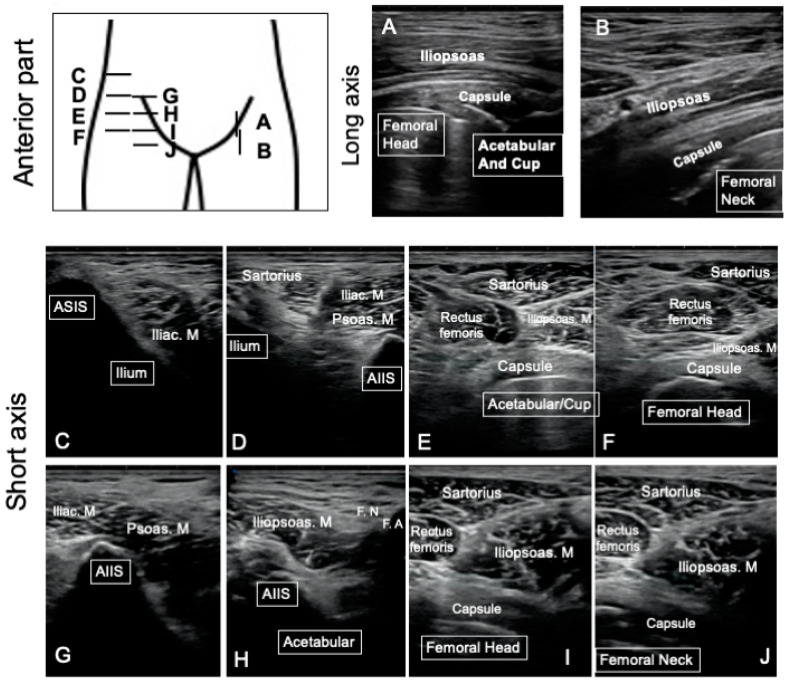
Representative sonography images of the anterior part in hip resurfacing patients. Periprosthetic hip soft tissues of the anterior part were assessed by longitudinal (**A**,**B**) and short axial (**C**–**J**) images. In the longitudinal images, the iliopsoas and capsule in front of the acetabular, cup, resurfacing head, and femoral neck were observed (**A**,**B**). In the short axial images (**C**–**J**), the iliac and psoas muscle, sartorius, rectus femoris, and capsule were observed using the anterior superior iliac supine (ASIS) (**C**), anterior inferior iliac supine (AIIS) (**D**,**G**,**H**), femoral nerve (F.N.), and femoral artery (F.A.) (**H**) as bony landmarks.

**Figure 2:**
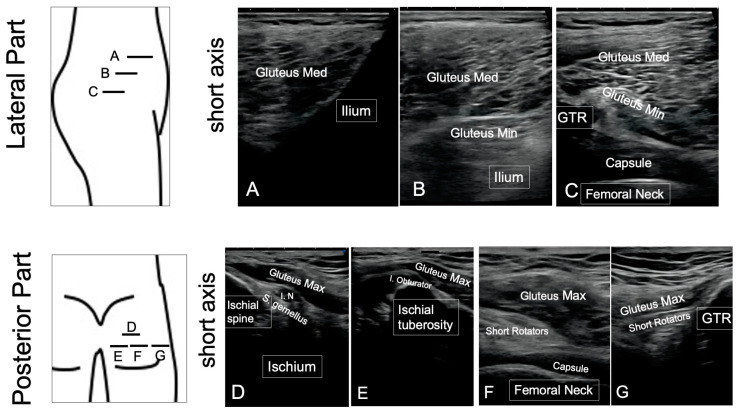
Representative sonography images of the lateral and posterior part in hip resurfacing patients. Periprosthetic hip soft tissues were assessed using short axial images in the lateral (**A**–**C**) and posterior part (**D**–**G**). In the lateral assessments, the gluteus medius and minimus were observed using ASIS and GTR as bony landmarks (**A**–**C**). In the posterior assessment, the gluteus maximus, short rotators, and capsule were observed using the ischial tuberosity and GTR as bony landmarks (**D**–**G**). Gluteus Med, gluteus medius; Gluteus Min, gluteus minimus; ASIS, anterior superior iliac supine; GTR, greater trochanteric; I.N., ischial nerve; S. gemellus, superior gemellus muscle; I. Obturator, internal obturator muscle.

The standardized protocol for ultrasonography assessments was conducted on the anterior, lateral, and posterior part of the hip. In terms of the protocol, the ultrasound screen commenced with an assessment of the anterior part in the supine position, the lateral part in the lateral position, and the posterior part in the prone position from the proximal part to the distal part (Figure 1). These periprosthetic assessments involved a careful observation of the joint capsule, iliopsoas, sartorius and rectus femoris in the anterior part; fascia tensor, gluteus medius, and gluteus minimus in the lateral part; and gluteus maximus and short rotators in the posterior part (Figure 1). Each periprosthetic muscle was observed using the same protocol and the following bony landmarks: the anterior superior iliac spine (ASIS), anterior inferior iliac spine (AIIS), and femoral artery for the anterior assessment (Figure 1); ASIS and the greater trochanter for the lateral assessment; and the GTR and ischium tuberosity for the posterior assessment (Figure 2). During assessment of the anterior part in the supine position, the distance from the resurfacing head implant to the iliopsoas muscle was measured in the long axis view to quantify the presence or absence of the mass region in the anterior part (Figure 1). All assessments were conducted from the height of the ASIS to the lesser trochanteric. SNiBLE (Konica Minolta, Tokyo, Japan) was utilized for these assessments except for the four cases requiring revision surgery for whom a LOGIQ 7 device (GE Healthcare, IL, USA) was utilized for the ultrasonography.

### 2.3. MRI

**Figure 3:**
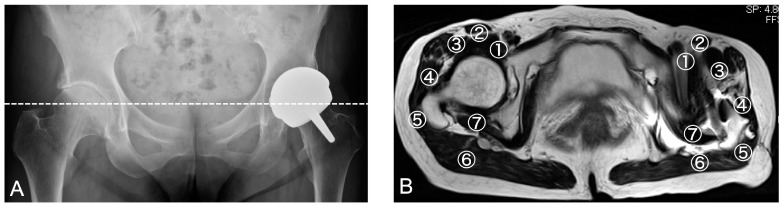
Representative hip resurfacing case assessed using X-ray and MRI. Apparent complications in this representative patient were not found by hip X-ray (**A**). The periprosthetic muscles consisted of 1. iliopsoas, 2. sartorius, 3. rectus femoris, 4. gluteus minimus, 5. gluteus midius, 6. gluteus maximus, and 7. short rotators which were observed by axial MRI (**B**) from the ASIS to lessor trochanteric level on T1 and T2 STIR images. If the contours of the targeting muscles cannot be observed, the muscle is regarded as unassessable.

MRI scans were performed using a 12 channel 1.5-T MR unit (MAGNETOM symphony; A Tim system, Siemens, Germany). Axial and coronal T1 sequence images were obtained with the following parameters: a repetition time (TR) of 500 ms, echo time (TE) of 8.3 min, echo train length (ETL) of 3, receiver band width (RBW) of 195 Hz/px, matrix of axial; 230 × 256 and coronal: 256 × 320, field of view (FOV) from 280 to 340 mm depending on the patient size, 1 excitation, and a slice thickness from 4 to 506 mm depending on the patient size. Axial and coronal STIR sequence images were also obtained under the following conditions: TR of 6000 ms, TE of 86 min, ETL of 15, RBW of 200 Hz/px, matrix of 224 × 320, FOV from 280 to 340 mm, and a slice thickness of 4–6 mm within the total acquisition time of 20 min. All MRI images were analyzed by the same surgeon (HC) separately from the ultrasonography. Each periprosthetic muscle was assessed by MRI using a cross sectional image of T1 and STIR from the height of the ASIS to the lesser trochanterics (Figure 3). In the patients that received multiple MRI assessments, the latest assessment, or the closest one to the occurrence of complications, was chosen for our investigation. The definitive diagnosis of a periprosthetic abnormality was made using MRI findings. The results from our assessments of periprosthetic abnormalities on MRI scans were then compared to the findings obtained using ultrasound.

### 2.4. Clinical Outcome

During the follow-up period, the Harris Hip Score (HHS) was noted for the assessment of hip function clinical scores. If there were no abnormal findings on the MRI or unusual pain (i.e., below 40 points in the HHS pain section), the patients were regarded as having made normal progress after the HRA. The diagnosis of ARMD was based on the detection of a pseudotumor on the MRI and through a histopathological assessment of intraoperative tissues with the exclusion of infection [17]. PJI was diagnosed based on cultures and histopathological assessment [18]. Any other abnormal hip pain was carefully assessed by clinical examination including ultrasonography-guided Xylocaine injection.

### 2.5. Statistical Analysis

All statistical analysis was performed with Prism 8 software (GraphPad Prism Version 8.4.3, CA). Statistical significance was determined by the Mann–Whitney test after confirming that the data were not normally distributed using the Shapiro–Wilk test. The accuracy of ultrasound for the detection of abnormal findings in the HRA cohort was determined by calculating its sensitivity and specificity in comparison with MRI findings. The visibility of periprosthetic muscles was compared between ultrasonography and MRI, and statistical analyses were conducted using the Fisher’s exact test. All tests were reported as significant if the *p* value was less than 0.05.

## 3. Results

**Figure 4:**
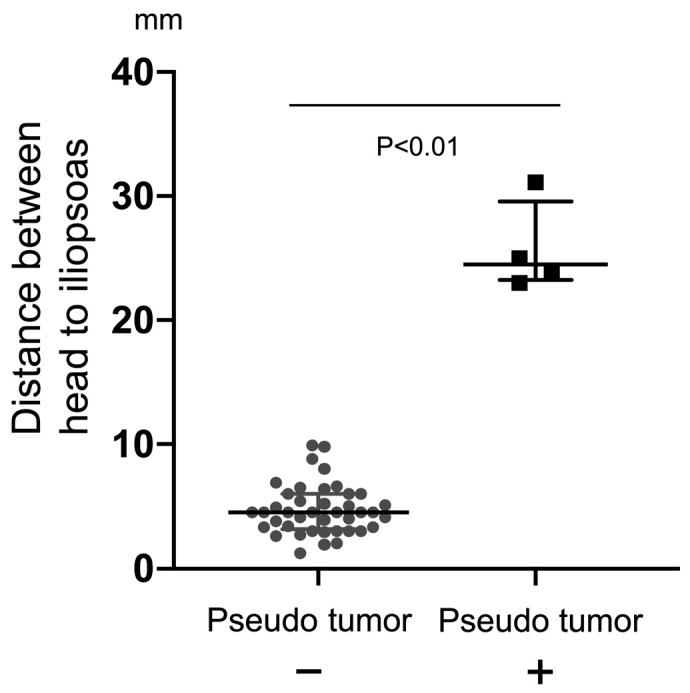
Comparison of the anterior distance between pseudotumor and non-tumor patients. The distances between the resurfacing head and the iliopsoas in the anterior part were measured in all HRA patients by ultrasonography. The median distance was significantly higher in the pseudotumor patients (4.5 mm vs. 24.5 mm, *p* < 0.01, Mann–Whitney test). Bar denotes median and interquartile range.

**Figure 5:**
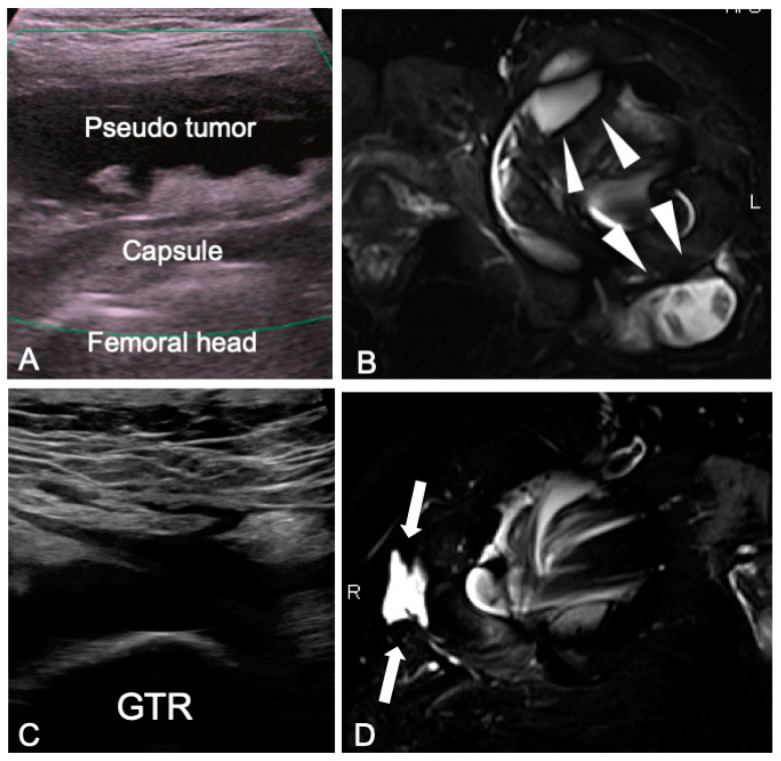
Representative abnormal images taken using ultrasonography and MRI in the hip resurfacing patients. (**A**,**B**): A pseudotumor was detectable in the anterior long axis of the hip by ultrasonography as a free echoic space above the femoral head (**A**). MRI provided information regarding the area of spread and lesion properties (white arrow heads) that is vital for the preoperative assessment of the pseudotumor (**B**). The trochanteric synovium was detected by ultrasonography in a patient with lateral thigh pain as a free echoic space located above the greater trochanter (**C**). MRI also provided information on local fluid collection (white arrows) at the lateral side of the greater trochanter (**D**).

The mean age of the current study’s patients was 50 years (range, 34–64 years). The mean Harris Hip Score was significantly improved from 55 points (mean pain score, 20 points) to 98 points (mean pain score, 43 points) at 1-year post-operation (Table 1) and to 96 points with a mean pain score of 41 points at the final follow-up. During the mean follow-up period of 8.6 years (range, 4.1–10.7), a periprosthetic abnormal mass region of a pseudotumor [19,20] was detected in four cases by both MRI and ultrasonography. Among these affected patients, two cases were diagnosed with ARMD and two with PJI (Table 1). All abnormalities were detectable by ultrasonography as apparent free echoic spaces in the anterior part of the implant (Figure 4 and Figure 5). The accuracy of ultrasonography for the detection of a pseudotumor thus had a sensitivity and specificity of 100%. The distance from the implant to the iliopsoas measured by ultrasonography in the ARMD or PJI cases was significantly longer than in the other cases (median lengths of 24.5 mm and 4.5 mm, respectively, *p* < 0.01; Figure 4).

**Figure 6:**
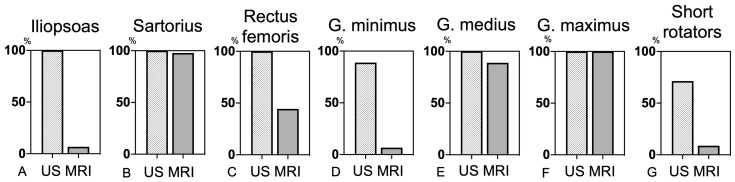
Visibility of periprosthetic muscles in the study cohort by ultrasonography and magnetic resonance image. In the anterior part, the iliopsoas, sartorius, and rectus femoris were detectable by MRI in 6.7%, 97.8%, and 44.4% of the HRA cases, although ultrasonography showed 100% of visibility of all these muscles (**A**–**C**). In the lateral part, the gluteus minimus and medius could be detected in 6.7% and 88.9% of the patients by MRI, and in 88.9% and 100% of these cases by ultrasonography (**D**,**E**). The gluteus maximus and short rotators were detectable in 100% and 8.8% of the patients by MRI and 100% and 71.4% of the patients by ultrasonography (**F**,**G**).

Among the 41 non-pseudotumor hips in our present HRA series, 4 cases were diagnosed with greater trochanteric bursitis through the detection of fluid accumulation on an MRI scan with sharp local pain recorded on the greater trochanter. In these four patients, a low echoic region around the greater trochanteric was observed on ultrasonography (Figure 6). In one case, iliopsoas impingement was diagnosed through clinical symptoms that included hip flexion pain which immediately improved after an ultrasonography-guided Xylocaine injection. No structural abnormality was observed on the iliopsoas by either MRI or ultrasonography in this case. Overall, both MRI and ultrasonography detected a periprosthetic abnormality in eight out of nine hips that manifested unusual pain after the HRA procedure.

In our assessments of periprosthetic muscles, MRI could produce a clear image of the iliopsoas in 6.7%, sartorius in 97.8%, rectus femoris in 44.4%, gluteus minimus in 6.7%, gluteus medius in 89%, gluteus maximus in 100%, and short rotators in 8.8% of the patients (Figure 6). These data indicated that the iliopsoas, gluteus minimus, and short rotators are not clearly detectable by either T1 or STIR MRI images in most HRA cases, as shown in Figure 3. Notably, however, ultrasonography enabled us to assess a complete and clear image of the iliopsoas, sartorius, rectus femoris, gluteus medius, and gluteus maximus with 100% visibility. However, neither the gluteus minimus nor short rotators could be clearly visualized in some cases, and the visibility was reduced to 88.9% and 72%, respectively (Figure 6). Hence, in comparison with MRI findings, ultrasonography showed greater utility in the assessment of the anterior and posterior parts of the periprosthetic muscles in HRA patients and thus presented the possibility of making a more precise assessment of periprosthetic abnormalities than MRI (Figure 6).

## 4. Discussion

Ultrasonography is an increasingly prevalent diagnostic modality in the orthopedic field due to advances made in the image resolution. In addition to its rapidity and convenience, an advantage of ultrasonography for the screening of abnormalities in THA patients has been its capacity to closely evaluate periprosthetic abnormalities that can be masked on an MRI or CT scan by metal halation. The early detection of implant related complications is vital in MOMTHA patients to avoid periprosthetic soft tissue damage due to ARMD [15,21,22]. In addition, abnormal periprosthetic reactions may be presenting through subtle symptoms [23]. As represented by ARMD, most implant-related complications originate in periprosthetic soft tissues. Therefore, periprosthetic areas need to be carefully assessed in MOMTHA cases in image screening. Previous studies have demonstrated the efficacy of ultrasonography in diagnosing ARMD and the utility of ultrasonographic classification for the prediction of revision THA, but no prior report has proposed a ultrasonographic screening protocol for periprosthetic soft tissue areas in HRA patients [7,8,9,16,24]. Hence, the major goal of our present study was to assess the accuracy of ultrasonography for this purpose using a standardized protocol that focused on the screening of abnormal reactions of periprosthetic soft tissue in HRA patients and a comparison with MRI findings.

In our present analyses, we unified our screening protocol to target the periprosthetic muscles. Because a unified record is vital for time course comparisons during a long-term follow-up period, we focused on bony landmarks of ASIS, AIIS, and femoral artery for the anterior assessment; ASIS and the greater trochanter for the lateral assessment; and the GTR and ischium tuberosity for the posterior assessment. Without these landmarks, operator-dependent assessments lack consistency in imaging and interpretation records. The advantage of our current protocol in this regard was that we used bony landmarks and surrounding periprosthetic muscle as guideposts that provided a good and consistent orientation of the hip joint. For example, in the anterior evaluations, ASIS and AIIS are good landmarks for identifying the iliopsoas and sartorius muscle, and the rectus femoris. Because these muscles always exist around the hip joint, their evaluation naturally involves observation of the anterior part of the hip joint. In the lateral evaluations, ASIS and the greater trochanter were landmarks for assessing the middle and gluteal muscles and are useful for observing the lateral side of the hip joint. In patients with obesity, posterior evaluations are often difficult, but the greater trochanter serves as an index for finding the greater gluteal muscle and external short rotators. The identification of the superior gemellus muscle which attaches to the ischial spine, or the internal obturator muscle that crosses the sciatic notch located just above ischial tuberosity, is an index for observations of the external short rotators and ischial nerve, although thick fat sometimes disturbs the identification of the external short rotator clear edges. In addition, by observing the ischial tuberosity from the greater trochanter, the posterior hip joint can be observed. The use of bony landmarks can provide reproducibility and accurate records, even in the obese patients. In addition, the measurement of the distance between the implant and viable muscle can provide a simple and brief assessment of a possible pseudotumor in HRA patients. In practice, the quantification of the anterior part is easily carried out by detecting the iliopsoas, although lateral and posterior assessments are still required for the overall evaluation of HRA complications [7,13].

The advantages of MRI are its ability to provide a macroscopic orientation, status, and property of a lesion that cannot be assessed with ultrasonography alone. In other words, MRI is useful for making a definitive diagnosis and lesion type classification, and for determination of the surgical area. However, a major drawback of MRI is that the metal artifact in HRA patients causes a significant loss of the periprosthetic area. The STIR images were tailored for these assessments in our HRA cases, but cannot ensure a qualified assessment of some parts of the periprosthetic area. In addition, the low sensitivity of MRI in detecting periprosthetic muscles indicates the possibility of an underestimation in detecting small inflammatory regions in the screening of asymptomatic patients [23]. For these reasons, we considered ultrasonography as a possibly better modality for identifying small legions. Another advantage of ultrasonography over MRI is its utility for conducting direct injections of Xylocaine for the detection of pain localization. The diagnosis of ARMD and PJI is important in the postoperative screening of HRA patients, but bursitis or tendinitis may be as prevalent in causing painful THA in these cases and are difficult to diagnose using MRI images alone [1]. Ultrasonography is useful, however, in diagnosing bursitis or tendinitis as represented by the detection of iliopsoas impingement using an echo-guided Xylocaine test in THA patients. On the other hand, ultrasonography screening still requires radiological assessments for the detection of bony abnormalities represented by osteolysis around the prosthesis, as these cannot be visualized by ultrasound.

A notable limitation of our present study was that we only analyzed a small population of HRA patients. Our screening protocol can be improved by assessing larger populations. Another limitation of our current analysis was the inability of ultrasound to assess the deep lateral and posterior muscle of the gluteus minimus and the short rotator in some obese patients due to the thickness of the fat layer, or, in some cases, due to changes in the soft tissue properties caused by the surgical intervention. Although these deep layer muscles were not able to be identified by MRI either, we believe that the use of a probe with a higher resolution and at a lower frequency will help resolve this limitation in the near future.

## 5. Conclusions

We have developed a unified ultrasonographic screening protocol that targets the periprosthetic tissues in HRA patients and provides a screening modality for the detection of ARMD, PJI, and bursitis that is as reliable as MRI and can more precisely detect periprosthetic muscles compared to MRI. Ultrasonography is, therefore, a feasible imaging modality for the routine screening of abnormal legions in HRA patients and our unified protocol can provide consistent assessments that may be vital in THA patients who require long-term follow-up evaluations after their operation.

## Figures and Tables

**Table 1:** Patient data before and after hip resurfacing arthroplasty.

Number of Hips (Patients): 45 (40)		Mean	Range
Age at surgery (years old)		50	34–64
Gender (hips)		Female: 35, Male 10	
Harris Hip Score (Points)	Pre-operative	Mean: 55 (pain: 20)	33–80 (10–30)
	Postoperative (at 1 year)	98 (pain: 43)	80–100 (30–44)
Follow-up period (years)		8.6	4.1–10.7
Complications during follow-up (Hips)		Adverse reaction metal debris: 2 Periprosthetic joint infection: 2 Greater trochanteric bursitis: 4 Iliopsoas impingement: 1	

## Data Availability

The datasets used and/or analyzed during the current study are available from the corresponding author on reasonable request.

## Abbreviations

THA, total hip arthroplasty; ARMD, adverse reaction to metal debris; MRI, magnetic resonance image; CT, computed tomography; HRA, hip resurfacing arthroplasty; ASIS, anterior superior iliac supine; AIIS, anterior inferior iliac supine; F.N., femoral nerve; F.A., femoral artery.
